# Cholinergic and noradrenergic aspects of Parkinson’s disease

**DOI:** 10.1093/brain/awae072

**Published:** 2024-04-04

**Authors:** Masud Husain

**Affiliations:** Oxford, UK

While neuropathological studies have long made it abundantly clear that Parkinson’s disease extends beyond degeneration of dopaminergic neurons, it has not been until relatively recently that *in vivo* studies have revealed the extent to which other neurotransmitter systems are compromised in the condition. Neuroimaging studies, in particular, have led the way to understanding how such changes might contribute to non-motor manifestations of Parkinson’s disease.

The evidence for cholinergic loss was already very compelling. The investigation reported by Schumacher and colleagues^1^ in a recent paper in *Brain* adds substantially to this evidence. They used the PET tracer ^11^C-methyl-4-piperidinyl propionate (PMP), which indexes cortical acetylcholinesterase activity, and also measured basal forebrain volume in 143 patients with Parkinson’s disease and 52 healthy individuals. Their key finding was that posterior basal forebrain volume was significantly correlated with PMP PET signal in temporo-parietal cortex. Further, these measures were related to cognitive function, both a global index and specific measures of memory and visuospatial ability. Indeed, participants who fulfilled the criteria for mild cognitive impairment (MCI) showed evidence for decreased overall cortical acetylcholinesterase activity.

A second study in *Brain,* performed by Crowley *et al.*,^2^ deployed vesicular acetylcholine transporter ^18^F-fluoroethoxybenzovesamicol (FEOBV) PET imaging in 101 patients with Parkinson’s disease. These investigators found that cortical acetylcholine signal was significantly associated with different domains of cognition, as were basal forebrain volumes and free water volume. In addition, they report that the basal forebrain metrics may mediate associations between cortical acetylcholine and cognitive performance.

Both these sets of results are consistent with a growing body of evidence that supports cortical cholinergic loss accompanying the transition to dementia in Parkinson’s disease.^3^ They also provide an important mechanistic rationale for the use of cholinesterase inhibitors in patients with Parkinson’s disease dementia. A recent study in *Brain Communications* reported the performance of patients when ON versus OFF (missing the morning dose) of rivastigmine, a widely used cholinesterase inhibitor.^4^ The results showed that being ON rivastigmine significantly improved attention, speeding up responses on a sustained attention task and improving performance on a working memory paradigm in a manner that suggested it improved visual attention. Restoring cholinergic tone may therefore have selective impacts on attentional deficits, but crucially not all aspects of cognitive impairment in Parkinson’s disease.^5^

Far less is known about noradrenergic disruption, although several lines of evidence suggest that it might be important.^6^ In this month’s issue of *Brain*, Laurencin and colleagues^7^ combined ^11^C-yohimibine (which binds to α2 adrenergic receptors) PET with neuromelanin sensitive MRI (to delineate the locus coeruleus). Patients with Parkinson’s disease had widespread reduction of α2 adrenergic receptors, including in the motor cortex, insula, thalamus and putamen. They also had diminished signal intensity in the locus coeruleus, which correlated with apathy and fatigue.

These findings concerning the relationship between the noradrenergic system and behavioural symptoms are particularly interesting. For example, atomoxetine, a noradrenaline reuptake inhibitor, may have a modulatory role on the relative precision of prior beliefs about action outcomes in patients with Parkinson’s disease and apathy.^8^ Participants in whom the locus coeruleus was smaller—and presumably atrophied—had a more positive change in their prior precision. Whether these findings would translate into clinically significant outcomes remains to be tested, but they add to a growing body of research which points to the use of non-dopaminergic drugs to treat some of the manifestations of Parkinson’s disease for which currently there are few options. Indeed, they beg the question why pharmaceutical companies show very little interest in pursuing cholinergic and noradrenergic targets with the vigour with which they seek to cure neurodegenerative diseases. I certainly know where I’d put my money.
